# Conditional Stat1 Ablation Reveals the Importance of Interferon Signaling for Immunity to *Listeria monocytogenes* Infection

**DOI:** 10.1371/journal.ppat.1002763

**Published:** 2012-06-14

**Authors:** Elisabeth Kernbauer, Verena Maier, Dagmar Stoiber, Birgit Strobl, Christine Schneckenleithner, Veronika Sexl, Ursula Reichart, Boris Reizis, Ulrich Kalinke, Amanda Jamieson, Mathias Müller, Thomas Decker

**Affiliations:** 1 Max F. Perutz Laboratories, University of Vienna, Vienna, Austria; 2 Ludwig Boltzmann Institute for Cancer Research (LBI-CR), Vienna, Austria; 3 Institute of Pharmacology, Centre for Physiology and Pharmacology, Medical University of Vienna, Vienna, Austria; 4 Institute of Animal Breeding and Genetics, University of Veterinary Medicine Vienna, Vienna, Austria; 5 Institute of Pharmacology and Toxicology, University of Veterinary Medicine Vienna, Vienna, Austria; 6 Biomodels Austria, University of Veterinary Medicine Vienna, Vienna, Austria; 7 Department of Microbiology and Immunology, Columbia University Medical Center, New York, New York, United States of America; 8 Twincore, Center for Experimental and Clinical Infection Research, Hannover, Germany; University of Toronto, Canada

## Abstract

Signal transducer and activator of transcription 1 (Stat1) is a key player in responses to interferons (IFN). Mutations of Stat1 cause severe immune deficiencies in humans and mice. Here we investigate the importance of Stat1 signaling for the innate and secondary immune response to the intracellular bacterial pathogen *Listeria monocytogenes* (Lm). Cell type-restricted ablation of the Stat1 gene in naïve animals revealed unique roles in three cell types: macrophage Stat1 signaling protected against lethal Lm infection, whereas Stat1 ablation in dendritic cells (DC) did not affect survival. T lymphocyte Stat1 reduced survival. Type I IFN (IFN-I) signaling in T lymphocytes reportedly weakens innate resistance to Lm. Surprisingly, the effect of Stat1 signaling was much more pronounced, indicating a contribution of Stat1 to pathways other than the IFN-I pathway. In stark contrast, Stat1 activity in both DC and T cells contributed positively to secondary immune responses against Lm in immunized animals, while macrophage Stat1 was dispensable. Our findings provide the first genetic evidence that Stat1 signaling in different cell types produces antagonistic effects on innate protection against Lm that are obscured in mice with complete Stat1 deficiency. They further demonstrate a drastic change in the cell type-dependent Stat1 requirement for memory responses to Lm infection.

## Author Summary

Signal transducer and activator of transcription 1 (Stat1) is an indispensable component of the cellular response to interferons (IFN) during immune reactions to pathogens. Stat1 deficiency leads to severe immune defects in humans and mice. The sensitivity of animals with complete Stat1 ablation to microbial pathogens prevented determining its contribution to various effector systems of the immune response. By way of tissue-restricted Stat1 ablation we now decipher the impact of Stat1 signaling in different cell populations on the innate and adaptive immune response to the intracellular pathogen *Listeria monocytogenes*. Our data highlight the importance of and requirement for IFNγ-activated macrophages for clearance of the pathogen during early phases of infection, and show a yet unanticipated detrimental role for T cell Stat1. During secondary responses the picture changes and Stat1 in T cells is crucial for proper clearance of *L. monocytogenes*. Likewise, Stat1 signaling in dendritic cells plays a fundamental role for adaptive immunity to *L. monocytogenes*. Exploring the local response to *L. monocytogenes* infection we reveal a role of Stat1 in shaping the cellular composition of inflammatory infiltrates. Furthermore, Stat1 deficiency in dendritic cells increases the proliferation of regulatory T cells, an effect likely to dampen the antibacterial response.

## Introduction

Signal transducer and activator of transcription (Stat1) is a central mediator of interferon responses in the immune system. Signals from type I (IFNα/IFNβ; IFN-I), type II (IFNγ; IFN-II) and type III (IFNλ, IFN-III) interferons employ receptor-associated Janus kinases (Jaks) to activate Stats by tyrosine phosphorylation [1], [2]. Gene transcription is induced and leads to a range of cellular changes, including anti-viral properties, growth inhibition, apoptosis and differentiation. Depending on the cellular context Stat1 can act as either a tumour-suppressor or promote oncogenesis [3], [4], [5]. The central character of Stat1 in signal transduction by the IFN receptors results from the importance of Stat1 homodimers for transcriptional regulation by IFNγ. Moreover, Stat1 forms the ISGF3 complex together with Stat2 and interferon regulatory factor 9 (Irf9). ISGF3 is the main player in transcriptional responses to both IFN-I and IFN-III. Consistent with its central role, Stat1 deficiency in mice recapitulates the lack of IFN-I, IFN-III and IFNγ responses and leads to high susceptibility to viral and bacterial infections [6], [7], [8]. The critical importance of Stat1 for resistance to infection is emphasized by mutations of the Stat1 gene in humans. Patients with various degrees of Stat1 loss-of-function present clinically with recurrent and often lethal mycobacterial and viral infections [9], [10], [11].

*Listeria monocytogenes* (Lm) is the causative agent of human listeriosis and a serious threat for the health of immunocompromised individuals. It is also a well-studied model organism to analyse cell-mediated immunity to intracellular pathogens. Innate protection critically depends on the activities of the cytokines interleukin (IL) 12 and IFNγ [12], [13]. This most likely reflects NK cell activation, IFNγ production and subsequent clearance of the bacteria by activated macrophages. Sterile immunity and immunological memory result from the development of CD8+ T cells [14], [15]. Stat1-deficient mice succumb to Lm during the early, innate phase of infection, strongly suggesting a dominant role for Stat1 in IFNγ-mediated macrophage activation [7]. As even very low numbers of Lm, even if attenuated, rapidly kill Stat1−/− mice it is difficult to study attributes of the innate response. For example, Lm replicates in a variety of non-hematopoietic cell types such as epithelial cells or hepatocytes and the contribution of Stat1 to bacterial clearance in these cell types is not known. Moreover, the impact of Stat1 on the generation of adaptive immunity and immunological memory is unclear [16], [17], [18], [19], [20]. In this regard the potential role of both IFN-I and IFNγ in the maturation and activation of dendritic cells [21], [22], [23] and the impact of both IFN types on the development of effector and memory CTL is of particular interest. Moreover, it has not been possible to investigate a potential contribution of macrophage activation to Lm clearance in secondary immune responses of mice lacking Stat1 in all tissues.

Further interest in cell type-specific Stat1 activities derives from the opposing effects of IFNγ and IFN-I on innate resistance to Lm. IFNγ-deficient mice show a similar susceptibility as Stat1−/− mice [24]. By contrast IFN-I receptor (Ifnar) deficient mice are protected from lethal Lm infections [25], [26], [27]. Suppression of protective innate immunity by IFN-I was suggested to result from increased T lymphocyte apoptosis and subsequent IL10-mediated immunosuppression [28]. Furthermore, infection of macrophages and DCs with Lm causes IFN-I dependent downregulation of the IFNγ-receptor, hence unresponsiveness to IFNγ [29]. IFN-I also sensitize infected macrophages *in vitro* to die from infection with Lm [30], [31].

To overcome the limitations posed by the exquisite sensitivity of Stat1−/− mice to infections with Lm or other pathogens we generated mice with floxed Stat1 alleles. Here we report that cell type-restricted Stat1 ablation reveals a striking dichotomy of immunological effects. Macrophage Stat1 produces protective innate immunity whereas the opposite is true for T lymphocytes. In secondary immune responses to Lm T lymphocyte and dendritic cell Stat1 signaling becomes protective, but Stat1 in macrophages does not contribute to clearance of bacteria.

## Results

### STAT1 in the hematopoietic compartment is crucial for host protection to *Listeria monocytogenes* infection

**Figure 1:**
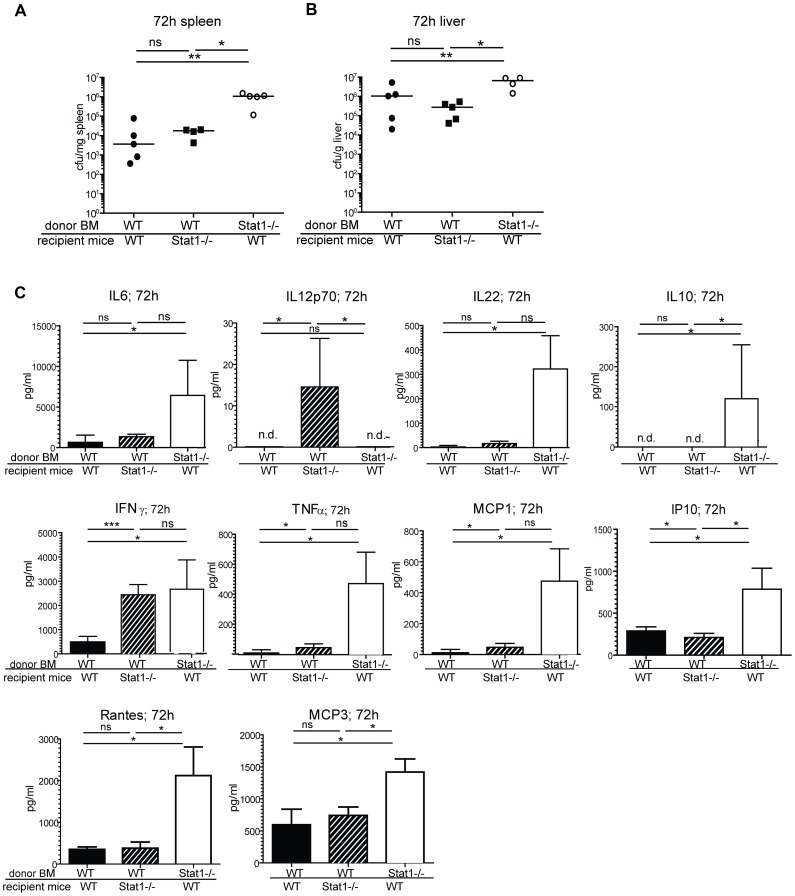
Role of STAT1 in the hematopoietic compartment. C57BL/6N (WT) and Stat1−/− mice were lethally irradiated and bone marrow of the respective donor mice was transferred into recipient mice. After engraftment mice were infected i.p. with 1×10∧5 Lm and the bacterial load of spleen (1A) and liver (1B) was determined and medians were calculated (n = 5). Statistical significance was determined using the Mann-Whitney Test. Serum of infected mice (n = 5) was collected and cytokines (IL6, IL12p70, IL22, IL10, IFNγ, TNFα, MCP1, IP10, Rantes, MCP3) were determined (1C). Means and standard deviations are shown.

To decipher the importance of Stat1 signaling for protective immunity to Lm in the hematopoietic and non-hematopoietic cell compartments we conducted adoptive transfer experiments. WT and Stat1−/− mice were lethally irradiated and bone marrow of either Stat1−/− or WT background was implanted in these mice. After 8 weeks the chimerism was examined in blood, spleen and liver showing an efficient implantation of the transferred bone marrow (supplemental figure S1). Bone marrow-chimeric mice were subjected to intraperitoneal infections with sublethal doses of Lm and 72 hrs later the bacterial burden in spleen and liver was determined (figure 1A, 1B). Compared to WT mice reconstituted with WT bone marrow, mice lacking Stat1 in non-hematopoietic cells showed a minor reduction of bacterial clearance, hence minor contribution of non-hematopoietic Stat1 to innate resistance. This suggests that hepatocytes, although representing an important niche for Lm multiplication [32], [33], are not protected by Stat1 signaling. By contrast mice lacking Stat1 in bone marrow-derived cells displayed a clear loss of resistance.

In addition to pathogen clearance we tested the impact of Stat1 deficiency on the systemic cytokine response. WT mice which received Stat1−/− bone marrow responded to infection with a systemic cytokine storm, i.e elevated serum levels of almost all measured cytokines and chemokines (IL6, IL22, TNFα, MCP1, IL10, Rantes, IP10 and MCP3; figure 1C). This is likely to reflect the increase in bacterial burden, hence a higher intensity of the innate response. Intriguingly however, the highest levels of IL12p70 were determined in the group of Stat1−/− mice that received protective WT bone marrow and had very similar bacterial loads as WT mice. This suggests that Stat1 of non-hematopoietic cells participates in the negative regulation of IL12 synthesis. In line with increased IL12, IFNγ production was elevated compared to WT. Likewise IFNγ was increased in mice with Stat1−/− bone marrow although IL12 levels were normal. Therefore, IL12 and IFNγ levels are not strictly correlated. In this situation IFNγ synthesis is most likely part of the cytokine storm as a consequence of high bacterial burden.

### Stat1 signaling in myeloid cells is essential, whereas Stat1 in T cells reduces innate resistance to *Listeria monocytogenes* infection

**Figure 2:**
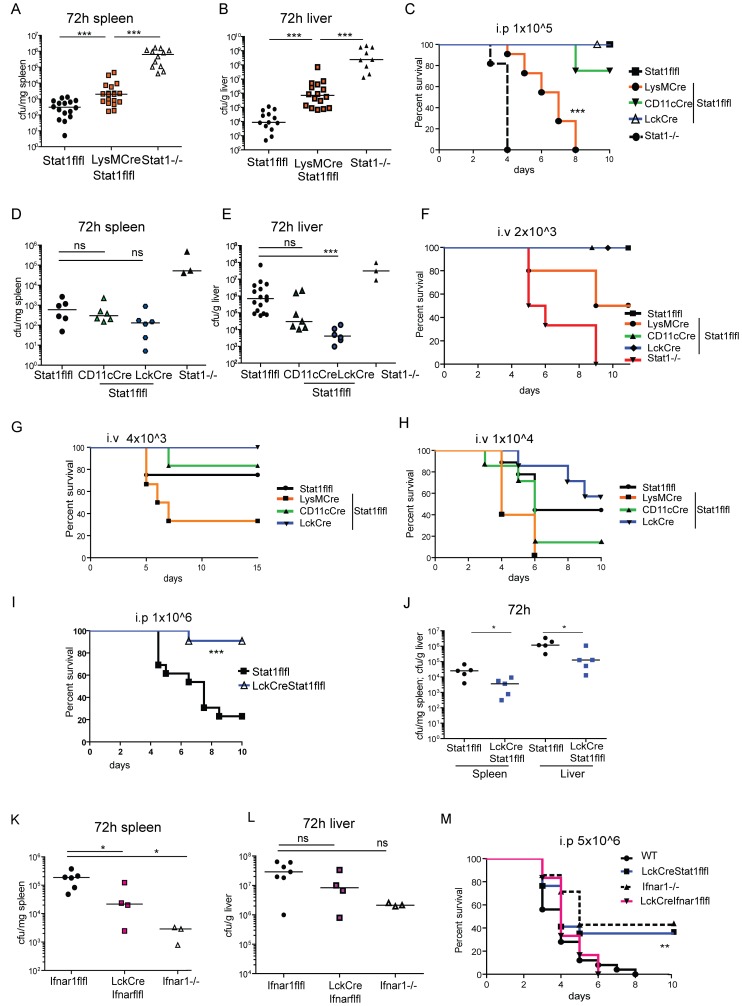
Bacterial load and survival in mice with cell-type specific Stat1 ablation. Stat1flfl, Stat1−/− and LysMCreStat1flfl mice were infected for 72 h with 1×10∧5 Lm i.p and bacterial load of spleen (A) and liver (B) was determined after 72 h or survival was monitored over 10 days (n = 11–14) (C). Stat1flfl, Stat1−/−, CD11cCreStat1flfl and LckCreStat1flfl mice were infected with 1×10∧5 Lm i.p and bacterial load was determined after 72 h in spleen (D) or liver (E). The survival of i.v. infected animals was monitored over 10 days with different doses of Lm; 2×10∧3 (F); 4×10∧3 (G), 1×10∧4 (H). Stat1flfl and LckCreStat1flfl mice were infected with 1×10∧6 Lm and survival was monitored over 10 days (n = 11–13) (I). LckCreStat1flfl and control mice (J) and LckCreIfnar1flfl and respective control mice (K; L) were infected with 1×10∧6 Lm and the CFU of spleen and liver was determined after 72 h. Survival was monitored for 10 days (n = 6–15) after infection with 5×10∧6 Lm i.p. (M). Representative results of at least two independent experiments are shown.

To further study the contribution of individual immunecompetent cells for the innate phase of Lm infection we analysed resistance to lethal infection and bacterial clearance after tissue-restricted Stat1 ablation. To determine the importance of Stat1 signaling in myeloid cells we used LysMCreStat1flfl mice, which delete predominantly in macrophages and neutrophils [34], [35]. These mice display a significantly reduced ability to clear even a low dose of Lm from spleen and liver (figure 2A, 2B) and hence succumbed to infection, whereas all WT mice survived the intraperitoneal infection (figure 2C).

To test the involvement of Stat1 to the immune response to Lm in other cell types of the immune system, mice with Stat1 deficiency in CD11c-positive cells, predominantly dendritic cells, but also subpopulations of NK cells and alveolar macrophages, were generated (CD11cCreStat1flfl, figure S2); [36], [37]. Mice lacking Stat1 in T cells were obtained by crossing Stat1flfl to LckCre mice (LckCreStat1flfl; figure S2). DC- and T cell- deleted mouse strains were subjected to a sublethal dose of Lm by intraperitoneal injection and bacterial loads in spleen and liver were monitored for the next three days (figure S3A, S3B). These mice did not show elevated numbers of Lm in spleen and liver at day three (figure 2D, 2E) or a significantly altered susceptibility to sublethal infection (2C).

Intravenous infection with Lm lead to the same outcome as intraperitoneal infection. Three different doses of Lm, ranging from sublethal to lethal referred to WT mice, where chosen to determine the response of the Stat1-ablated mice (figure 2F–H). Increased sensitivity to infection was seen when myeloid cells lacked Stat1, whereas CD11cCreStat1flfl animals behaved similar to WT. Strikingly, LckCreStat1flfl mice displayed increased resistance to high infectious doses. This consequence of T cell specific Stat1 ablation was similarly observed following intraperitoneal infection with a higher than LD50 inoculum of Lm. Lack of activity of T cell Stat1 resulted in both increased survival of the animals and an enhanced clearance of the bacteria from spleen and liver (figure 2I, 2J).

IFN-I signaling in T cells was previously shown to reduce the clearance of Lm upon infection in the spleen [26]. To examine whether the enhanced survival in LckCreStat1flfl mice was due to a lack of IFN-I signaling, we analysed the bacterial load and survival in mice with IFN-I receptor deficiency in T cells (LckCreIfnarflfl). We noted a significantly lower number of splenic Lm in LckCreIfnarflfl mice compared to WT mice at high doses of infection, whereas the number of bacteria in the liver was not significantly reduced (figure 2K, 2L). Mice with complete Ifnar1 deficiency showed a clearly better ability to contain Lm infection than mice lacking Ifnar1 only in T cells. The increased ability of LckCreIfnarflfl mice to clear bacteria in the spleen did not result in a higher rate of survival compared to WT mice, whereas complete Ifnar1 deficiency did (figure 2M). This result suggests that the increase in resistance produced by the absence of T cell Stat1 cannot be entirely explained on the basis of the lack of IFN-I signaling in T cells.

### Stat1's contribution to splenocyte apoptosis reflects IFN-I signalling

**Figure 3:**
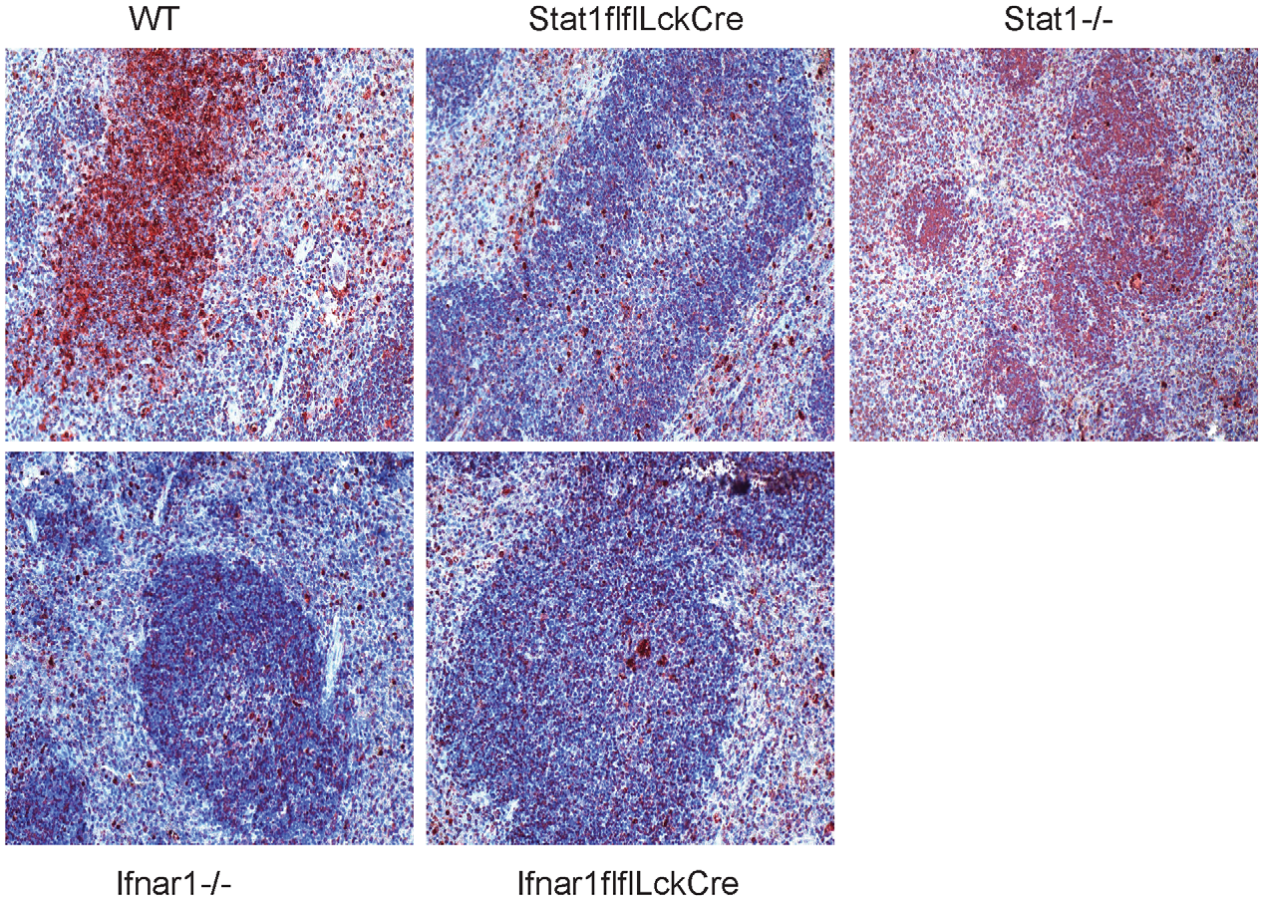
Apoptotic cell death in the spleen. WT, LckCreStat1flfl, LckCreIfnar1flfl, Ifnar1−/− and Stat1−/− were infected with 1×10∧6 Lm and spleens were isolated 48 h after infection. TUNEL positive cells are visible in dark red; hematoxyline counterstaining indicates the structure of the spleen (3).

TUNEL staining of spleen cells two days after i.p infection with Lm produced the expected large number of apoptotic cells in WT mice [26], which was strongly reduced in both LckCreStat1flfl and LckCreIfnar1flfl mice (figure 3). Since the decrease in cell apoptosis resulting from either Stat1 or Ifnar1 ablation was highly similar, the additional protection of LckCreStat1flfl mice from Lm infection is not due to a lesser rate of infection-induced apoptosis in Stat1-deficient T cells.

To further clarify the difference of LckCreStat1flfl and LckIfnar1flfl we isolated splenocytes of these genotypes and appropriate controls (WT, Stat1−/−, Ifnar1−/−) and infected them *in vitro* for two days with Lm at MOI 10. Subsequently we analysed the supernatant of these cultures for T cell cytokines. Stat1 deficiency in T cells lead to increased production of IL4 and IL17 and a clear suppression of IFNγ. In contrast, Ifnar1 deficiency in T cells did not decrease production of the signature cytokines under study, but increased the amounts of IFNγ, IL17 and IL10. The data indicate a Th population-independent negative regulation of T cell activation by IFN-I and demonstrate the strong influence of Stat1 on the generation of Th1 cells through its target gene T-bet [38] (figure S4). Thus, Ifnar or Stat1 ablation in T cells impact differently on the generation and function of Th cell populations *in vitro*.

### Systemic cytokine levels in mice with cell specific Stat1 ablation after *Listeria monocytogenes* infection

**Figure 4:**
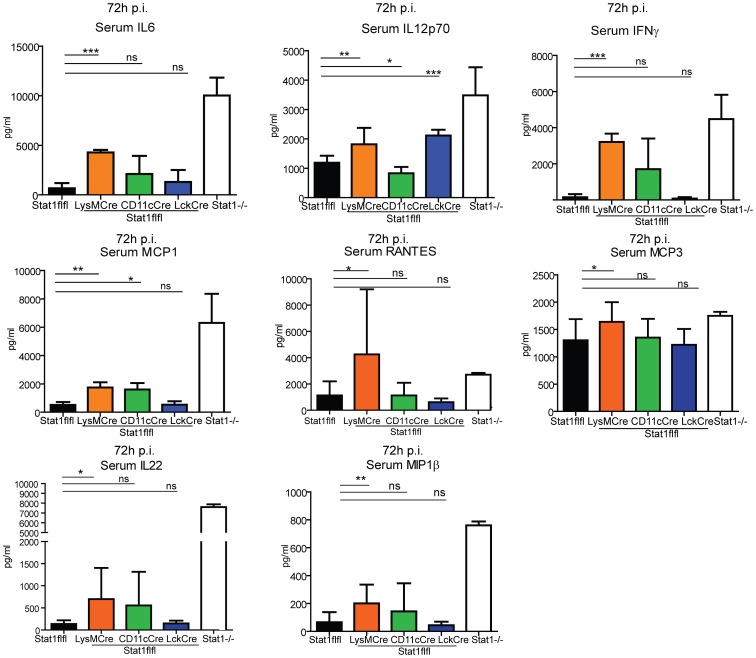
Analysis of serum cytokines in mice with cell-type specific Stat1 ablation 72 hrs after infection with Lm. Mice with Stat1 ablation in different cell compartments were infected with 1×10∧5 Lm and serum was collected over the course of three days after infection. Indicated cytokines (MCP1, IL6, IFNγ, IL12p70, MCP3, Rantes, MIP1β, IL22) were analysed (4). Mean values of cumulative data out of two experiments (n = 8) are depicted with standard deviations. Significant differences are indicated using asterisks.

Examination of systemic cytokine/chemokine levels demonstrated that mice lacking myeloid Stat1 signaling show increased levels of IL6, IL12p70, MCP1, MCP3, IL22, MIP1β, Rantes and IFNγ in their serum, similar to but not as dramatic as complete Stat1 deficiency (figure 4). As these mice have strongly elevated numbers of pathogens in their organs the increase in inflammatory cytokines may again reflect an increased activity of the innate immune system. Alternatively, increased cytokine production could also result from the loss of Stat1-mediated gene repression as reported for IL6 [39]. The function of Stat1 as both a transcriptional activator and repressor is well documented [40]. Both functions require binding to GAS sequences [41], but the detailed mechanisms are not understood.

Stat1 signaling in CD11c+ cells had a very selective impact on the levels of systemic cytokines, showing elevated levels of MCP1 compared to WT mice but interestingly, lower amounts of IL12p70. By contrast, higher levels of IL12p70 were detected in the serum of mice lacking Stat1 signaling in T cells, despite an equal bacterial load. The levels of TNFα and IL10 were too low to be detectable at this dose of infection.

### Organ damage in mice with cell type-restricted ablation of Stat1 signaling after *Listeria monocytogenes* infection

**Figure 5:**
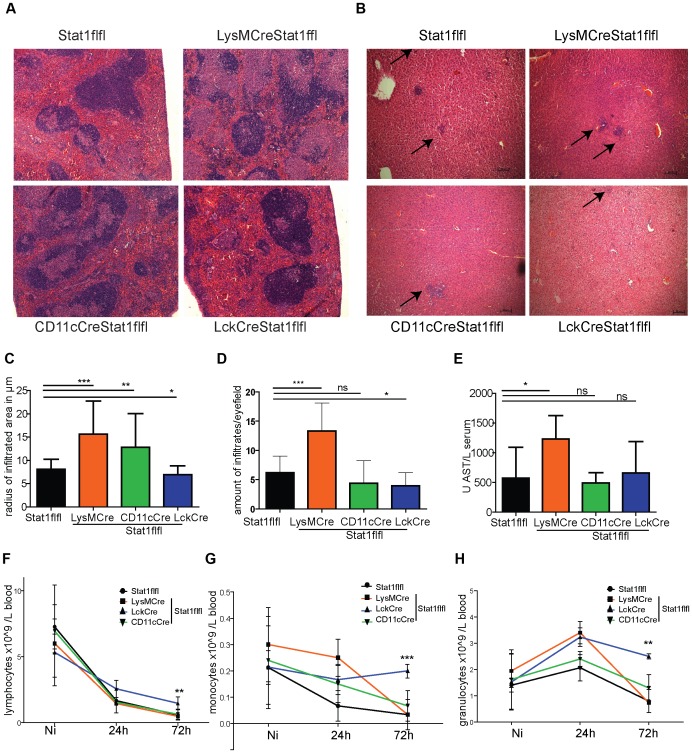
Organ damage and blood cell populations after 72 h of infection. Spleen (5A) and liver (5B) sections of animals infected with Lm for 72 hrs were stained with H&E, arrows point to infiltrates in the liver. The amount of infiltrates and the radius of the infiltrated area was measured in µm of three individual mice in three microscopic fields (10×) using the Zeiss AxiVision LE software and depicted in (5C, 5D). One representative picture is shown. Amino aspartate transferase levels were analysed in the serum of indicated mice (n = 6) infected with Lm for 72 h (5E). Numbers of lymphocytes (5F), monocytes (5G) and granulocytes (5H) were determined in blood samples of uninfected mice (Ni) or of mice after infection for 24 hrs or 72 hrs (n = 3–6) using a vet haematology counter. Data from two independent experiments are shown and statistical significances are indicated with asterisks where applicable.

Spleens (figure 5A) and livers (figure 5B) of infected animals with conditional Stat1 gene ablation were analysed using H&E staining to determine the severity of inflammation two days post-infection. In keeping with the loss of innate resistance, mice lacking Stat1 in myeloid cells showed a severe pathology of the spleen with increased lymphocyte depletion [42]. In the liver the radius of the inflammatory infiltrate area, classified as microabscess [43], correlated with the increase of bacteria found in this organ (figure 5B, 5C). In addition, the numbers of micro-abscesses correlated with bacterial burden, as lack of Stat1 in myeloid cells increased the area of infiltrates in the liver. Whereas CD11c+ cell-specific ablation of Stat1 led to significantly bigger areas of infiltrates compared to WT (figure 5C), the amount of infiltrates (figure 5D) and bacteria in the liver was not significantly enhanced compared to WT (figure 2E). Mice with T cell-restricted Stat1 gene deletion showed smaller infiltrate areas compared to the WT, again reflecting the protection of these mice from infection.

Liver failure may significantly contribute to the lethality of Lm infection [44]. To assess liver damage, we measured the amount of circulating amino aspartate transferase (AST), an enzyme released from damaged hepatocytes and readily measurable in serum samples [45] (figure 5E). LysMCreStat1flfl mice displayed strongly elevated levels of AST, indicating massive liver damage. No significant differences in AST were found in all other animals/genotypes compared to WT animals.

In addition to organ damage we examined the immune status of Stat1-ablated mice by analysing the composition of blood leukocytes. Mice lacking Stat1 in T cells had the highest numbers of circulating immune cells in their blood 72 h after infection (figure 5F, 5G, 5H). This is consistent with the notion that the reduced number of Lm in organs, coupled with reduced numbers of apoptotic cells led to a diminished recruitment of blood leukocytes. Additionally this result may indicate a defect in Stat1 regulated synthesis of T cell-derived chemokines.

### STAT1 signaling regulates cellular influx to local sites of inflammation

**Figure 6:**
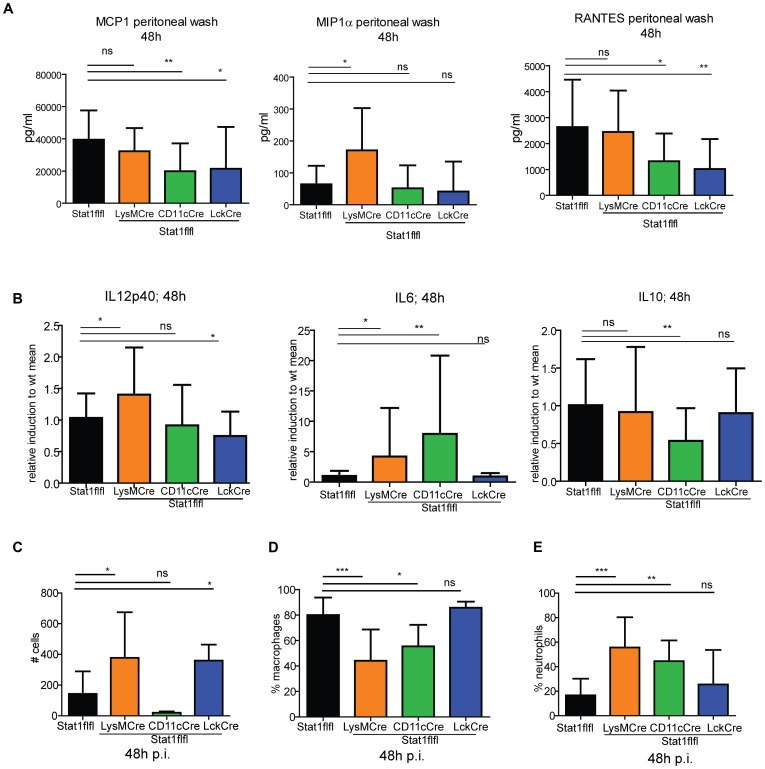
Peritoneal inflammation 48 hrs after infection. Chemokines (MCP1, Mip1α, Rantes) were determined in the peritoneal lavage fluid of mice (n = 6) after 48 hrs of infection (6A). Peritoneal exudate cells were isolated 48 h after i.p. infection with 1×10∧6 Lm, adherent cells were enriched and RNA prepared and subjected to qPCR for IL12p40, IL6 and IL10 (6B). Data from 3–5 experiments (n = 10–15) are pooled and means with standard deviation are shown. 48 h after i.p infection peritoneal exudate cells were isolated and cytospins stained with Wright-Giemsa solution. The amount of cells (6C), the percentage of macrophages (6D) and the percentage of neutrophils (6E) were counted and depicted (n = 9).

The results shown in figures 1 and 2 emphasize the importance of Stat1 mediated macrophage activation. In spite of this, mice completely devoid of Stat1 cleared Lm less well than LysMCreStat1flfl animals. This result could be explained by incomplete ablation of the Stat1 gene in macrophages although our recent inspection of macrophages demonstrates deletion with very high efficiency [35]. Alternatively or additionally, therefore, the difference between LysMCreStat1flfl and Stat1−/− mice may reflect shaping of the innate immune response by Stat1 signaling in several different leukocyte populations. In our infection model the peritoneum is the site of immediate exposure of innate cells to the bacterial pathogen that initiates a local inflammatory response. To determine the degree to which cell type-specific Stat1 signaling determines this local immune response, we first analyzed the local chemokine/cytokine milieu in the peritoneal cavity over the course of the first three days of infection (figure S6). The most striking differences between genotypes were observed at day 2 (figure 6A). Absence of Stat1 in myeloid cells increased MIP1α MCP1 and Rantes amounts at day two and three compared to WT. MCP1 and Rantes were decreased upon CD11c-Cre-mediated Stat1 ablation at day two, but the levels of these chemokines recovered and exceeded WT levels at day three. T cell-specific Stat1 ablation lead to a decrease in Rantes levels at day two after infection, at day three the amount of the tested chemokines reached WT level. Examination of the pro- and anti-inflammatory cytokine gene expression patterns of adherent peritoneal macrophages isolated from infected mice indicated a small but significant role of myeloid cell Stat1 in the negative regulation of IL12 (figure 6B). Remarkably, Stat1 signaling in T cells was required for full IL12p40 expression. In keeping with the aforementioned negative regulation by Stat1 a more profound effect was noted with regard to IL6 production that was markedly upregulated upon STAT1 deficiency in either myeloid cells or the CD11c+ population. The CD11cCreStat1flfl genotype was unique in producing an adherent cell population with reduced IL10 production. Together with the systemic analyses shown in figure 1 and 2 our data suggest that peritoneal macrophages are major producers of IL6 and IL12. Lack of Stat1 signaling in CD11c+ dendritic cells or inflammatory monocytes may stimulate macrophages to produce excess amounts of IL6 and decreased amounts of IL10.

To determine whether the altered peritoneal chemokine/cytokine levels changed the cell recruitment, we isolated peritoneal exudate cells two days after intraperitoneal Lm infection and analysed the cell composition by Wright-Giemsa-stained cytospins and flow cytometry (figure 6C–E). Myeloid cells together constitute >95% of the peritoneal exudates in WT mice. In animals lacking Stat1 in DC reduced numbers of leukocytes were recruited, however neutrophils were increased at the expense of macrophages (figures 6C–E). Thus, Stat1 signaling in CD11c+ DC regulates monocyte/macrophage migration to the inflamed peritoneum. Mice with myeloid Stat1 ablation showed an increased influx of total peritoneal leukocytes with a similar tendency to reduce monocytes/macrophages and increase neutrophils. Finally, the absence of Stat1 signaling in T cells caused a strong increase in the amount of immune cells travelling to the peritoneum without altering their composition.

Together the data characterizing the peritoneal inflammatory response suggest a profound impact of Stat1 in different cell types on the cytokine milieu and on leukocyte composition. This may explain in part why myeloid cell-restricted Stat1 ablation does not fully reproduce the loss of bacterial clearance observed upon complete Stat1 gene deletion.

### Stat1 expression in DC and T cells regulates adaptive immunity to *Listeria monocytogenes*

**Figure 7:**
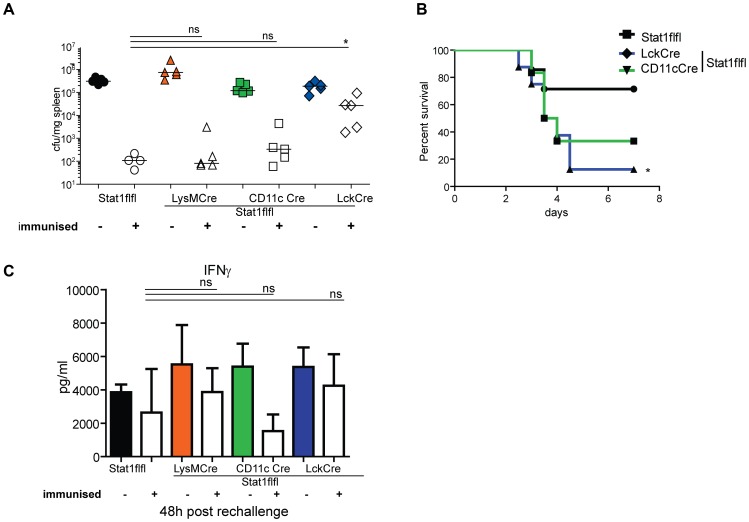
Adaptive immunity to *Listeria monocytogenes* in mice with tissue-restricted Stat1 ablation. Immunised and naïve mice were infected i.v. with 1×10∧5 Lm and bacterial load was determined 48 h after infection (n = 4–5) (7A) or survival of 7 mice of each genotype was monitored (7B). Serum of immunised and naïve mice was analysed for the presence of IFNγ (7C). One representative result of at least two independently performed experiments is shown (n = 4–5).

To analyse the impact of Stat1 signaling in different cell populations on establishing adaptive immunity against Lm, we applied an immunisation and challenge protocol to the respective conditional knockout mice. Under these conditions mice lacking Stat1 signaling in T cells failed to clear Lm from the spleen (figure 7A). Accordingly, an increased percentage of LckCreStat1flfl mice succumbed to infection compared to WT mice (figure 7B). Immunized mice lacking Stat1 in CD11c+ cells showed a slight impairment in clearing splenic Lm, yet the impact on survival was almost as pronounced as in mice lacking Stat1 in T cells. Myeloid Stat1 did not contribute to the establishment of adaptive immunity to Lm as bacterial clearance after immunisation was as strong as in WT mice.

Overall systemic cytokine levels were generally lower than those found after infection of naïve mice. Stat1 deficiency in CD11c+ cells caused a selective reduction of systemic IFNγ that may contribute to the reduced ability to raise adaptive immunity to Lm (figure 7C). The levels of IFNγ in mice lacking Stat1 signaling in T cells were equally high as in naïve mice. Given the reduced ability of Stat1−/− T cells to generate the Th1 lineage [38] (figure S4) this may reflect IFNγ production by cells other than Th1 or, alternatively, low numbers of Th1 cells developing in absence of Stat1 may produce higher IFNγ amounts due to the lack of the negative regulation Stat1 imposes on the IFNγ gene [46].

**Figure 8:**
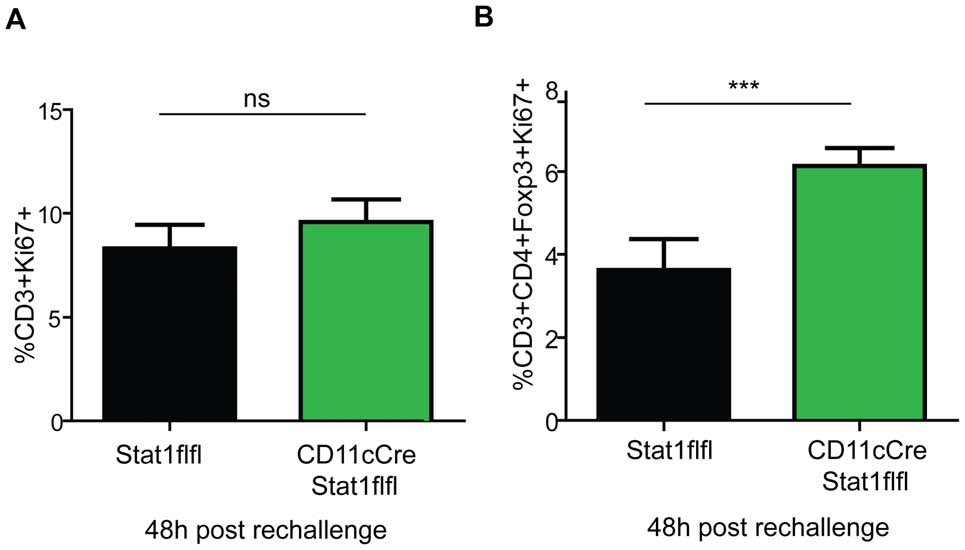
Dendritic cell Stat1 regulates adaptive immunity. Splenocytes of immunised and rechallenged Stat1flfl and CD11cCreStat1flfl mice were isolated and evaluated for proliferating CD3+ cells (8A) and Tregs (CD3+, CD4+, FoxP3+, Ki67+) (8B). Means and standard deviations of one representative experiment out of two independently performed experiments are shown with 5 mice per group.

To further analyse the immunisation defects in CD11cCreStat1flfl mice, we investigated T cell responses after immunisation. Proliferation of splenic CD3+ T cells showed no significant differences (figure 8A). However, examination of the Treg population (CD4+Foxp3+) revealed an enhanced proliferative response in the spleens of mice with CD11c+-restricted Stat1 ablation (figure 8B). As regulatory T cells represent only a minor percentage of total splenic T cells it is not surprising that the difference in proliferation went unnoticed when analyzed in the context of total CD3+ T cell cells. The data suggest a contribution of DC Stat1 to the control of proliferation of a small proportion of antigen-specific Treg.

## Discussion

Studies in gene-modified mice and with cells from human patients suffering from recurrent infectious disease have unequivocally established the central importance of Stat1 for the establishment of protective innate immunity to viral and nonviral pathogens [6], [7], [47]. This includes Lm, the bacterial pathogen studied here. Conditional gene targeting allowed us to examine whether there is a uniform immunological impact of Stat1 across different cell types. Furthermore, we were able to investigate the importance of Stat1 signaling in the same cell types for the development of acquired antibacterial immunity.

Clearance of intracellular bacterial pathogens is caused either by a microbicidal effector mechanism of the infected cell or indirectly through CD8+ T cell-mediated cytolysis. Lm infects a variety of different cell types *in vitro*, either by active invasion or phagocytosis [48], [49]. In infected mice the pathogen replicates in both hematopoietic cells, predominantly macrophages, and non-hematopoietic cells amongst which the hepatocytes form a major niche [32], [33]. To our surprise Stat1 signaling provides non-hematopoietic cells with little effector potential, posing the question how Listeria are killed in these cell compartments particularly before the influx of antigen-specific CTL. One possibility is the death of infected hepatocytes and the subsequent phagocytosis of the cell contents including bacterial cargo by phagocytic cells of the innate immune system [32]. Subsequent sterile clearance most likely requires the development of CTL and active lysis of infected cells [50], [51], [52]. The importance of clearing Lm in the liver is underscored by our findings that the death of mice with different Stat1 genotypes correlated well with the inflammatory infiltrate in this organ and with the hepatotoxicity caused by infection.

LysMCre mediated gene deletion occurs predominantly in macrophages and granulocytes [34]. A recent report shows that the contribution of neutrophils to the immune response against Lm is surprisingly small [53] and, given the short half life of granulocytes, Stat1-dependent transcriptional response to IFN is unlikely to enhance their microbicidal activity. Therefore our data are consistent with the previous notion that IFNγ and Stat1 cause macrophage activation and that activated macrophages represent a dominant innate anti-listericidal effector mechanism of the innate immune response [54], [55]. Indeed, our data reveal the essential role of this cell type for clearance of Lm. That said the clearance deficit of mice lacking macrophage Stat1 was significantly lower than that of Stat1−/− mice. In this regard inspection of the local immune reaction elicited by intraperitoneal infection with Lm. showed that besides macrophages T cells and particularly CD11c+ cells shape the inflammatory environment by regulating chemokine production and cell influx. CD11c+ cells in this situation are likely to represent inflammatory DC that arise from inflammatory monocytes [56]. Although lack of Stat1 signaling in non-hematopoietic cells or DC does not per se affect survival and clearance of infection, it may synergize with the Stat1 deficiencies of other cell types to produce the more severe outcome of the complete Stat1 knockout. Increased Lm replication in Stat1−/− compared to LysMCreStat1flfl mice caused a more severe cytokine storm that is likely to be one cause of their accelerated death.

While the amount of serum cytokines generally followed the severity of infection in different Stat1 genotypes, IL12 was the exception because it was strongly increased in mice with Stat1-deficiency outside the hematopoietic compartment that were able to cope with infection nearly as well as WT mice. This finding reveals negative regulation of IL12 synthesis by a non-hematopoietic cell. IL10 is a negative regulator of IL12 production [57] and its synthesis can be suppressed by IFNγ [58]. However, IL10 is considered to be a product of hematopoietic cells [59]. The nature of the suppressive cell type and the mechanism of IL12 suppression will require further investigation.

The exacerbation of infection by Stat1 in T lymphocytes is particularly intriguing. Unanue and colleagues demonstrated the T cell response to IFN-I as a mechanism underlying their adverse effect [26], [28]. While our data confirm that the IFN-I response of T cells indeed reduces bacterial clearance, Ifnar1-deficiency in this cell type alone does not reproduce the consequences of the complete Ifnar1 knockout and it does not cause the robust effect of T cell-specific Stat1 deletion. Hence, T cells indeed inhibit protective innate immunity to Lm, but the effect of Stat1 goes beyond IFN-I signaling. Furthermore, additional cell compartments must contribute to the suppressive effect of IFN-I, as suggested by the comparison between infected Ifnar−/− and LckCreIfnar1flfl mice. At present we do not fully understand how Stat1 signaling in T cells reduces innate immunity to Lm. Clearly, it increases apoptosis of splenic cells, and the comparison to Ifnar-ablated cells suggests this results from the activity of type I interferons. However, T cell Stat1-mediated loss of innate protection goes beyond IFN-I effects on splenocyte apoptosis. In line with the results shown in figure S4, skewed CD4+ T cell differentiation causing a reduction of IFNγ-producing Th1 cells and a concomitant increase in Th2 and Th17 cells may be a contributing factor. Importantly, Ifnar1 deficiency in T cells increases IFNγ production when Lm antigens are presented by wt APC *in vitro*. At present we do not know whether T cell differentiation is a decisive factor within the first three days of infection in mice. Systemic cytokine profiles of LckCreStat1flfl animals showed little change with respect to WT mice in this period. Systemic IL10 levels were below the detection limit, leading us to assume that reduced clearance does not result from global immunosuppression. In addition, bacterial multiplication might be enhanced by the reported suppressive activity of IFN-I on IFNγ receptor expression [29].

Infection of immunized mice with Lm caused a drastic change in the consequences of Stat1 signaling in cell types of the immune system. Most importantly Stat1 activity in T cells was now required for protective immunity. Antigen-specific CTL are critical effectors for adaptive immunity to Lm [15], [52]. Our data provide the first genetic proof that Stat1 signaling in both T cells and DC is required for acquired resistance, in addition to showing that IFNγ-activated macrophages are dispensable once memory lymphocytes have been produced. The essential stimulus for Stat1 signaling in T cells is unclear. IFN-I appear to be dispensable for both CD4+ and CD8+ T cell development during Lm infection [60], [61]. Furthermore, a study analyzing CTL development in IFNγ-deficient animals infected with very low numbers of Lm shows that IFNγ is not essential for protective CTL-mediated immunity [51]. Possibly IFNγ contributes to protective CTL memory when infection occurs with a high infectious dose. Therefore, Stat1 may increase the efficacy of memory CD8+ T cell responses.

Defective Stat1 signaling in CD11c+ DC also reduced protection by the adaptive immune system upon secondary challenge with Lm. This is consistent with our recent finding that immunization with Stat1−/− DC caused a strongly diminished CTL response to Ova peptide [62] and with reports by others that Stat1−/− DC fail to elicit protective immunity to *Leishmania major* [63]. IFN-I and Stat1 reportedly support DC maturation and activation [64], [65]. Data in the literature thus suggest a defect of Stat1-deficient DC to present antigen to T cells. In line with this IFN-I were reported to stimulate the ability of DC to cross-present antigen [23]. In our experiments the lack of DC Stat1 affected survival of mice more than splenic clearance of bacteria, which argues against a general defect in generating effector CTL. Moreover, activation of both CD4+ and CD8+ T cells to the levels found with wt cells occurred *in vitro* when Stat1−/− DC were used as antigen presenters (Figure S5). In a mouse model of graft versus host disease Ma and colleagues noted an increased proliferation of FoxP3+ regulatory T cells upon transfer of Stat1−/− splenocytes into irradiated hosts [66]. Prompted by this finding we tested whether Stat1 deficiency in DC might similarly cause increased Treg proliferation in Listeria-infected mice. Indeed we noted that the proportion of proliferating FoxP3+ cells was about two-fold higher in spleens from infected mice with a CD11cCreStat1flfl genotype. It is therefore possible that an increased number of antigen-specific regulatory T cells suppresses effector T cells and thus reduces the immune response to Lm.

In summary, cell type-restricted ablation reveals a fascinating complexity of Stat1's regulatory power for the development of both innate and adaptive immune responses to Lm.

## Materials and Methods

### Mice and bacteria

Animal experiments were discussed and approved by the University of Veterinary Medicine Vienna institutional ethics committee and carried out in accordance with protocols approved by the Austrian law (BMWF-68.205/0204-C/GT/2007; BMWF-68.205/0210-II/10b/2009, BMWF-68.205/0243-II/3b/2011). Bacteria were prepared for infection as described previously [67]. For infection, Lm LO28 were washed with PBS and injected intraperitoneally (i.p) or intravenously (i.v) of 8- to 10-week-old sex and age matched C57BL/6N (WT), Stat1flfl (B6.129P2-Stat1^tmBiat^, [35]), LysMCre Stat1flfl (B6.129P2-Lyz2^tm1(cre)Ifo^/J-Stat1^tmBiat^ [34]), CD11cCre Stat1flfl (B6.Cg-Tg(CD11c-Cre)-Stat1^tmBiat^ [68]), LckCre Stat1flfl (B6.129P2-Tg(LckCre)-Stat1^tmBiat^ LckCre [69]), Ifnar1−/− (B6.129P2-IfnaR1^tm1^) [70], Stat∧−/− (B6.129P2-Stat1tm1), Ifnar1flflLckCre (B6.129P2-IfnaR1^tm1^-Tg (LckCre) [71] mice at the respective dose. The infectious dose was controlled by plating serial dilutions on Oxford agar plates. The survival of mice was monitored for 10 days, and data were displayed as Kaplan-Meier plots. For determination of bacterial loads of liver and spleen mice were killed at the indicated time points. The respective organs were isolated and homogenized in PBS. Serial dilutions of the homogenates were plated on BHI plates and incubated at 37°C for 24 h. For immunisation mice were injected i.p with 1×10∧6 attenuated Listeria (ΔActA). After 2–3 weeks mice were infected i.v with 1×10∧5 Lm.

### Cytokine analysis

For cytokine analysis mice were bled via the retro-orbital sinuses and serum was collected and stored at −80°C. Using the FlowCytomix system (ebioscience) concentrations of indicated cytokines (IFNγ, IL6, IL10, IL12p70, Mcp1, Mcp3, Rantes, GMCSF, Mip1α, Mip1β, IP10, IL22, TNF α) in 25 µl of serum were measured.

### Peritoneal exudate cell isolation

For isolation of peritoneal macrophages mice were infected for 48 h with 5×10∧6 LO28 i.p. Mice were sacrificed, the peritoneum was flushed with two times 10 ml of DMEM and cells were harvested by centrifugation and plated on 6 well plates. After 2 h adherent cells were washed with PBS and RNA was prepared for Real Time PCR analysis. The composition of total peritoneal exudate cells was examined using Wright-Giemsa staining of cytospins. Composition of adherent cells were analysed by flowcytometric analysis (F4/80-APC, CD11b-PE, CD3-FITC (BD biosciences), Ly6C-PerCP (ebioscience)). Chemokines and cytokines were measured using the FlowCytomix system after flushing the peritoneum with 1 ml of DMEM.

### RNA isolation, cDNA synthesis and Real Time PCR

RNA was isolated using the Nucleospin II kit (Macherey and Nagel) according to protocol. Reverse transcription was accomplished using RevertAid (Fermentas). The Real Time PCRs were run on an Eppendorf cycler. After correction for the housekeeping gene Gapdh, every sample was calculated to the mean of WT mRNA levels. The following primer sequences were used (all 5′-3′): IL6: for TAGTCCTTCCTACCCCAATTTCC; rev TTGGTCCTTAGCCACTCCTTC; IL10: for GGTTGCCAAGCCTTATCGGA; rev ACCTGCTCCACTGCCTTGCT; IL12p40: for TGGTTTGCCATCGTTTTGCTG; rev ACAGGTGAGGTTCACTGTTTCT; GAPDH: for CATGGCCTTCCGTGTTCCTA; rev GCGGCACGTCAGATCCA.

### Flowcytometric analysis

Spleens were isolated after indicated timepoints and single cell suspensions were prepared using a 80 µm cell strainer. After red blood cell lysis cells were stained for CD3-PE, CD4-FITC, CD8-APC, CD11b-PerCP, Gr1-PE, FOXp3-APC, Ki67-PerCP (all BD bioscience). For intracellular staining cells were fixed and permeabilised using the FoxP3 staining kit (ebioscience)

### Histology

Mouse organs were fixed with 4% paraformaldehyde over night, paraffin embedded and 4 µm sections were prepared using a microtome. Hematoxyline and eosin staining (H&E) were performed using standard protocols. The radius of the infiltrate was measured using the Zeiss Axioplan software. For TUNEL staining, sections of spleens were stained using the TUNEL-POD kit (Roche) according to protocol. Additionally, sections were blocked for endogenous peroxidase activity in methanol with H_2_O_2_ and after proteinase K treatment blocked with 5% normal goat serum to reduce background staining. TUNEL enzyme and POD conversion was applied as described, and AEC+ high sensitivity chromogen (Dako) was used as a HRP substrate. Subsequently, sections were counterstained with hematoxyline.

### AST measurement

Aspartate amino transferase concentrations were measured in mouse serum using a COBASc11 analyzer (Roche).

### Adoptive transfer

WT (Ly5.1 and C57BL/6) and Stat1−/− animals were lethally irradiated with 8,2 Gy for 17 minutes and engrafted with 5×10∧6 bone marrow cells of respective genotypes by i.v injection. After 6 weeks engraftment was analysed in blood, spleen and liver by flow cytometry using the antibodies Ly5.1-FITC, Ly5.2-PE, CD11b-PerCP (BD biosciences).

### Blood lymphocyte population

Mice were bled via the retro-orbital sinuses in tubes coated with EDTA and the cellular composition was measured using a vet haematology analyzer V sight (A. Menarini diagnostics).

### Statistical analysis

Bacterial loads of organs were compared using the Mann-Whitney test; mRNA expression data and cytokines levels were analysed with the Students t test. For both the GraphPad Software was used. Asterisks describe the significances as follows: * p≤0,05; **p≤0,01; ***p≤0,001

## Supporting Information

Figure S1 **Bone marrow chimerism and blood lineage analysis.** Percentage of Ly5.1 positive leukocytes in the blood and Ly5.1+ and CD11b+ cells in spleen and liver. For bone marrow Ly5.2 positive cells are shown due to a large proportion of Ly5.1 and Ly5.2 negative cells. Percentages of positive cells are indicated in the figure (S1). Representative plots of a Stat1−/− mouse reconstituted with WT (Ly5.1) bone marrow are shown.
(EPS)

Figure S2 **Deletion efficiency of CD11c and LckCre in splenic DC and T cells.** Splenic dendritic cells (S2a) and splenic T cells (S2b) were isolated, respectively, from CD11cCreStat1flfl, LckCreStat1flfl, and control mice (S2b) and subjected to Western blot analysis for Stat1N and pan-erk.
(EPS)

Figure S3 **Time course of bacterial burden in mice with cell type-restricted Stat1 ablation.** Stat1-ablated and control mice were infected i.p with 1×10∧6 Lm and bacterial load of spleen (S3A) and liver (S3B) over the course of three days was determined. Medians of cumulative data from 2–3 experiments are depicted.
(EPS)

Figure S4 ***In vitro*** **infection of splenocyte cultures.** Splenocytes were isolated from WT, Ifnar1−/−, Stat1−/−, LckCreStat1flfl and LckCreIfnar1flfl mice and infected *in vitro* with Lm at a MOI of 10. 48 h after infection the supernatant was harvested and analysed for IFNγ, IL4, IL17 and IL10. Means of cumulative data of two individual experiments are shown with standard deviations.
(EPS)

Figure S5 **Time course of serum cytokines after Lm infection.** The respective Stat1 ablated animals were infected with 1×10∧6 Lm and respective cytokines were measured in 25 µl of serum at day 1, 2 and 3 after infection. Means of cumulative data from 2–3 experiments are shown with standard deviation. Significances are indicated with asterisks.
(EPS)

Figure S6 **Time course of peritoneal chemokines after Lm infection.** The peritoneum of mice infected for 48 h with 1×10∧6 Lm was flushed with 1 ml of DMEM. Chemokines were measured in 25 µl of the peritoneal wash. The means of cumulative data from 2 experiments are shown with standard deviation.
(EPS)

Figure S7 **Stat1−/− dendritic cells can stimulate T cell proliferation.** WT mice were immunised with ΔActA Lm and splenic T cells were isolated from these animals 2–3 weeks later. Splenic dendritic cells were isolated from WT and CD11cCreStat1flfl animals, pulsed over night with heat-killed Listeria (HKL) and admixed to the CFSE labelled T cells at different ratios. Unstimulated, CFSE labelled T cells were used as a control. Dilution of T cell CFSE was determined 8 days after stimulation. Means of percent divided (CFSElo) CD8+ Tcells and CD4+ Tcells are depicted of one representative experiment out of three.
(EPS)

Text S1 **Supplemental methods.**
(DOC)
