# Supplementary material for: Conditional Stat1 Ablation Reveals the Importance of Interferon Signaling for Immunity to Listeria monocytogenes Infection
Source: PLoS Pathog. 2012 Jun 14;8(6):e1002763. doi: 10.1371/journal.ppat.1002763 (PMC3375314; doi:10.1371/journal.ppat.1002763)
Supplement: Text S1 — Supplemental methods. (DOC) [file ppat.1002763.s008.doc]

Text S1

Supplemental method for S2

Western Blot analysis of MACS purified splenic DC and T cells was performed following standard protocols. Fluorophore-linked secondary antibodies (LI-COR) were used for detection on an Odyssey infrared imaging system (LI-COR). Antibodies against Stat1N (Cell Signalling) and against pan-erk (BD biosciences) was used.

Supplemental method for S4

Spleens were isolated of the respective gene targeted mice, single suspensions were prepared and red blood cell lysis was performed. 5x10^5 splenocytes were seeded per well in a 96 well round bottom plate in RPMI + 10% FCS and kept at 37°C with 5% CO2. Cells were infected with a multiplicity of infection (MOI) of 10 with Lm LO28. After 1 hour plates were centrifuged, supernatant was carefully removed and medium was changed to RPMI +10% FCS + 50µg/ml Gentamicin to kill extracellular bacteria. After two hours of infection the medium was changed again to RPMI +10% FCS + 10µg/ml Gentamicin. The supernatant was harvested 48h after infection and analysed for T cell derived cytokines using the ebioscience FlowCytomix kit.

Supplemental method S7

Stat1flfl mice were immunised with 1x10^6 Lm ΔActA i.p and rechallenged with 1x10^5 Lm LO28wt i.v. Two days after rechallenge the spleens were isolated and T cells were purified using the MACS pan-T cell isolation kit II (Miltenyi) according to protocol. The T cells were stained in 1µM CFSE at 37°C for 15 minutes and the reaction was stopped with FCS on ice. 2x10^5 T cells were seeded into 96 well round bottom plates in RPMI +10% FCS. Splenic dendritic cells of Stat1flfl or Stat1-/- mice were isolated using CD11c microbeads (MACS, Miltenyi) and pulsed over night with HKL (MOI 10). Dendritic cells were added to the T cells at different ratios as indicated. Dilution of CFSE in T cells was monitored after staining for CD3-APC, CD4-PE and CD8-PerCP (BD bioscience) on a FACS Calibur machine at day 8. CFSE stained T cells were left alone as a control.
